# Validation of AI-based software for objectification of conjunctival provocation test

**DOI:** 10.1016/j.jacig.2023.100121

**Published:** 2023-05-30

**Authors:** Yury Yarin, Alexandra Kalaitzidou, Kira Bodrova, Ralph Mösges, Yannis Kalaidzidis

**Affiliations:** a Practice for ENT und Allergology, Dresden, Germany; b Max Planck Institute for Molecular Cell Biology and Genetics, Dresden, Germany; c ClinCompetence Cologne GmbH, Cologne, Germany

**Keywords:** Allergy, conjunctival provocation test, deep learning, artificial intelligence (AI)

## Abstract

**Background:**

Provocation tests are widely used in allergology to objectively reveal patients’ sensitivity to specific allergens. The objective quantification of an allergic reaction is a crucial characteristic of these tests. Because of the absence of objective quantitative measurements, the conjunctival provocation test (CPT) is a less frequently used method despite its sensitivity and simplicity.

**Objective:**

We developed a new artificial intelligence (AI)-based method, called AllergoEye, for quantitative evaluation of conjunctival allergic reactions and validated it in a clinical study.

**Methods:**

AllergoEye was implemented as a 2-component system. The first component is based on an Android smartphone camera for screening and imaging the patient’s eye, and the second is personal computer–based for image analysis and quantification. For the validation of AllergoEye, an open-label, prospective, monocentric study was carried out on 41 patients. Standardized CPT was performed with sequential titration of grass allergens in 4 dilutions, with the reaction evaluated by subjective/qualitative symptom scores and by quantitative AllergoEye scores.

**Results:**

AllergoEye demonstrated high sensitivity (98%) and specificity (90%) as compared with human estimation of allergic reaction. Tuning cutoff thresholds allowed us to increase the specificity of AllergoEye to 97%, at which point the correlation between detected sensitivity to allergen and specific IgE carrier–polymer system class becomes obvious. Strikingly, such correlation was not found with sensitivity to allergen detected on the basis of subjective and qualitative symptom scores.

**Conclusion:**

The clinical validation demonstrated that AllergoEye is a sensitive and efficient instrument for objective measurement of allergic reactions in CPT for clinical studies as well as for routine therapy control.

Provocation tests in allergology play an especially important role in the clinical cases of patients who have unclear allergic symptoms unrelated to a specific season or multiple skin reactions and carrier–polymer system (CAP) test results (eg, patients with perennial allergy such as house dust mite allergy or polyallergy).

Compared with other provocation tests, the conjunctival provocation test (CPT) is simpler, without compromising accuracy and sensitivity.^1^^,^^2^ In the past few years, the CPT has been used for diagnosis as well as for therapy control, and it is becoming a crucial instrument of clinical studies.^3^, ^4^, ^5^ However, despite the CPT's 100-year history,^3^^,^^6^ its role is still limited on account of its main drawback, namely, the subjective nature of the evaluation of outcomes. Nevertheless, the CPT has the same level of clinical importance as the nasal provocation test, which includes an objective instrumental evaluation of outcomes.^1^^,^^3^^,^^7^ Multiple clinical studies have demonstrated a high level of concordance between these 2 methods.^8^, ^9^, ^10^, ^11^

Until now, the main means of evaluating the CPT has been the summation symptom score (SSS).^2^ The SSS is the sum of the patient's categorically and ultimately subjective estimations of allergy symptoms (such as eye itching, irritation, and tearing) and eye redness subjectively estimated by medical staff (observers) carrying out the test.^3^^,^^6^^,^^11^

To overcome this drawback of the CPT, several approaches have been proposed for quantification of CPT outcomes.^4^^,^^12^, ^13^, ^14^ The readout of quantitative CPT methods is based on measurement of eye redness on digital images. The redness quantification can be subdivided into 2 approaches. The first approach is based on calculation of the apparent area of vessels in the sclera.^12^^,^^15^ Therefore, vessels were contrasted and segmented, after which the relative area of sclera covered by segmented vessels was taken as the redness score. However, the partial observation of the sclera and different eyeball orientations toward the camera on sequential observations could degrade the method's accuracy. Additionally, diffuse redness, which is related to the widening of capillaries that could not be resolved on nonmicroscopic images, is not captured by this method. The second approach calculates the redness for each pixel.^4^^,^^14^ The mean redness of the sclera^4^ or histogram of redness distribution^14^ is then used as a redness score. In this case, it must be noted that there are multiple definitions of redness,^4^^,^^16^^,^^17^ which could lead to different sensitivities of the method. The crucial problem of per-pixel redness estimation is its dependence on the reproducible color balance of the environment illumination and the automated white balance of digital images. The limited application of all of the aforementioned methods is due either to semiautomated image analysis,^4^^,^^12^^,^^18^ absence of automated correction for illumination color changes (white balance),^14^ or both.^4^^,^^12^^,^^14^^,^^18^

In medicine, the deep learning approach is applied in diagnostics, image reconstruction in radiology modalities, emergency care devices, etc.^19^, ^20^, ^21^ However, its use in allergology is still limited. We recently developed a new approach for the qualitative evaluation of CPT results for diagnostics of allergic rhinoconjunctivitis and implemented it in a high-throughput artificial intelligence (AI)-based software platform called AllergoEye, which comprises deep neural networks for automated image analysis and symptom evaluation.^17^ For the implementation of AllergoEye in clinical practice, this article demonstrates the results of the clinical validation and efficiency of AllergoEye in a cohort of patients with grass pollen allergy.

## Methods

### Approvals and ethics

The study was approved by the ethics committee of the State Chamber of Physicians of Saxony, Germany (identification no. EK-BR-111/21-1). The study was conducted in accordance with local regulations, the International Conference on Harmonization of Technical Requirements for Registration of Pharmaceuticals for Human Use (International Conference on Harmonization–Good Clinical Practice), and the Declaration of Helsinki.^22^ Patients signed informed consent forms before any study procedures were performed.

### Study design

This open-label, prospective, monocentric study was carried out in the Practice for Otorhinolaryngology and Allergology by Dr Yury Yarin (Dresden, Germany) in 2021. Female and male patients (N = 41) between 18 and 75 years of age were included in this study. The study of these patients with grass pollen allergy was performed outside the grass pollen season and consisted of 2 visits. Patients were included in the study only after they had signed the informed consent form (before any study procedures were performed). For participation in the study, patients were required to have a history of moderate or severe seasonal allergic rhinoconjunctivitis with or without seasonal controlled asthma during the 3 previous grass pollen seasons. To objectively ensure the existence of allergy as a criterion for inclusion in a study, a skin prick test and testing for the presence of grass pollen–specific IgE (sIgE) antibodies were performed. A wheal diameter of at least 3 mm for a grass pollen allergen solution and level of sIgE antibodies higher than 0.01 kU/L were the main necessary conditions for study inclusion. The exclusion criteria were adapted from the guidelines for daily practice^3^ (see the Supplementary Materials in the Online Repository at www.jaci-global.org).

During the first screening visit, allergic anamneses were collected and the inclusion and exclusion criteria were checked. Next, a skin prick test was performed and a blood sample was taken for antibody testing. During the second visit, the CPT was performed and estimated by summation symptoms scores (SSS),^18^ which were calculated at the end of the test by medical personnel.

Simultaneously with the CPT procedure, the results of the patient’s reactions to each applied dilution were additionally recorded by AllergoEye. The results of the CPT and AllergoEye platform were entered into a merged database and statistical analysis was performed.

### Titrated CPT procedure

Standardized lyophilized grass group allergen extracts for CPT with a concentration of 30 histamine equivalent potency units per milliliter (HEP/mL) (supplied by Laboratorios LETI S.L. [Madrid, Spain]) were used. Reconstitution of the CPT stock solution was performed by drawing 4.8 mL from a vial containing 5.0 mL (SD ± 0.2 mL) of solvent and dispensing into a vial containing grass extract. The CPT was performed in accordance with the following protocol: at the first step of the CPT, a negative control solution (diluent without allergens) is applied to the right eye. In the event of a negative reaction after 5 minutes, an allergen solution is applied stepwise to the left conjunctival sac in predefined increased concentrations (1:1000, 1:100, and 1:10) and stock solution with 5-minute intervals between each application. The symptoms are documented on a standardized form^18^ (see Table I in Fig E3 in the Online Repository at www.jaci-global.org). Four symptoms (tearing, itching, irritations, and redness) are analyzed. Each of these symptoms is estimated and categorized on a scale from 0 to 3, with 0 indicating no reaction, 1 indicating mild reaction, 2 indicating moderate reaction, and 3 indication strong reaction. The SSS is obtained by summing the scores for all 4 subjective (itching, irritation, and tearing) and objective (redness evaluated by medical staff) symptoms and used to obtain a value ranging from 0 to 12. The protocol is stopped when medical personnel detect redness of the treated eye or the maximum allergen concentration has been achieved.

### AllergoEye: Measurements and protocol

**Fig 1:**
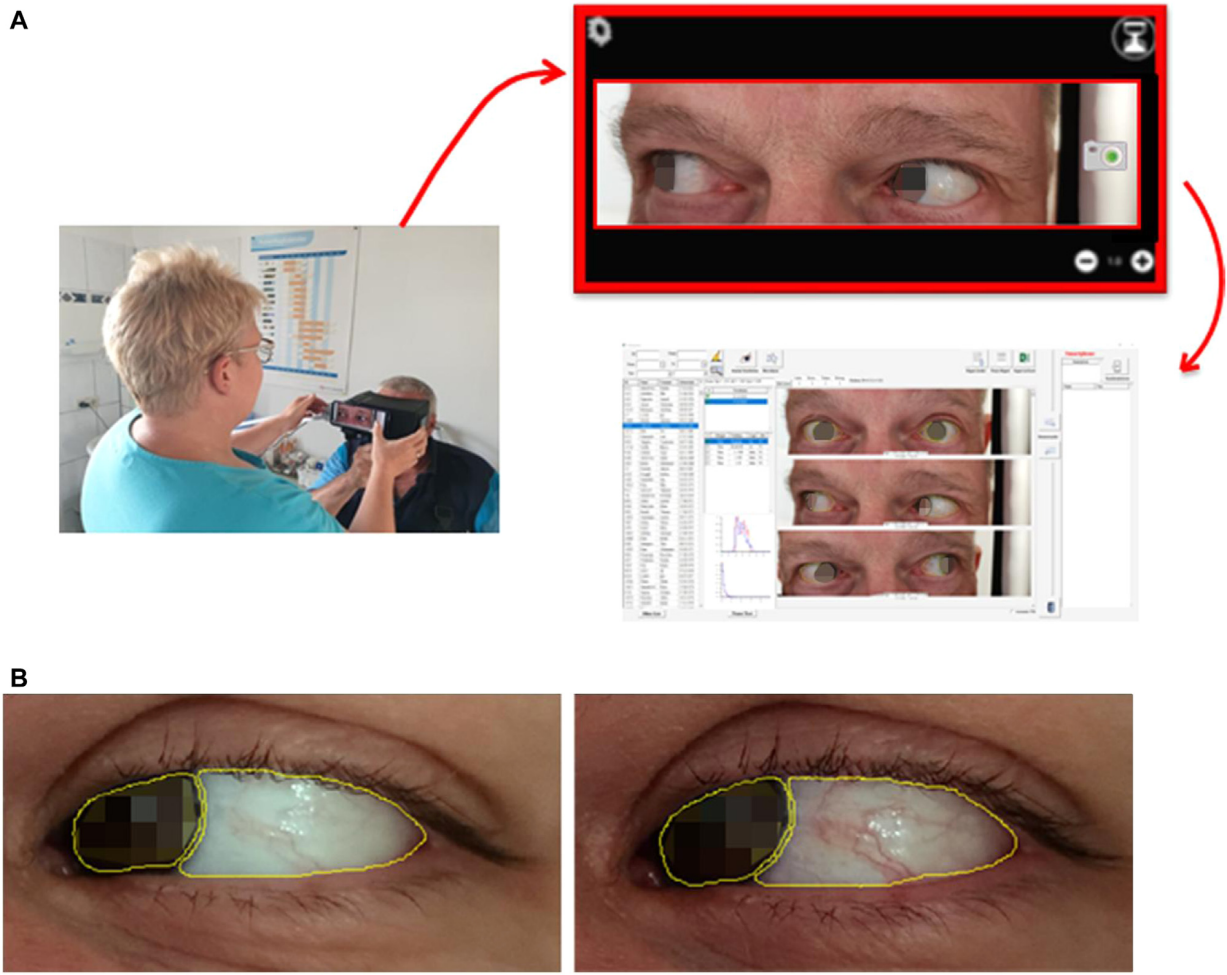
Workflow of PCT evaluation by AllergoEye. **A,** (*left*) A nurse takes images of the patient’s eyes by smartphone using the AllergoEye mobile app (*right*), after which the image (*top*) is wirelessly transferred to the PC-based AllergoEye application (*bottom right*), and the analysis result is transferred to the mobile application for control. **B,** The sclera and iris are segmented by neural network (*contoured in yellow*). Sclera redness before (*left image*) and after (*right image*) provocation is shown.

During the CPT, which we described earlier in this article, both of the patient's eyes are imaged 5 minutes after each provocation. The provocation is performed on the left eye with sequentially increased concentrations of the grass CPT solution. Control images are also taken before the test and after treatment of the right eye with a control solution (test solution without allergen). The eyes are imaged in 3 positions (looking straight ahead, to the right, and to the left) for maximum coverage of the sclera. To control the illumination conditions, the images are taken with a special mask (Fig 1, *A*) with continuous white light–emitting diode (LED) illumination. At the end of the test, the images are sent to a personal computer (PC), where the redness is evaluated by using the AllergoEye software.

### AllergoEye: Technical description

AllergoEye was implemented as a 2-component system. One part is based on an HONOR 20 Pro Android smartphone (Huawei, Shenzhen, China) and uses the built-in high-resolution photocamera for imaging the patient eye’s reaction to the allergen (Fig 1, *A*). The second part is a PC-based software system, including the patient database, the communication module for exchanging data with multiple smartphones, and a deep neural network for image analysis and redness evaluation. In short, the patient's data and measurement details (allergen, dilution, and exposure time) are transferred from the PC to the mobile phone. The nurse acquires images of the patient's 2 eyes and wirelessly transfers them to the PC. There, the deep neural network is used to recognize and segment the iris and sclera. Additional control of coupling of iris and sclera as well as the presence of 2 open eyes was performed to exclude segmentation artifacts. We have proposed a new method of per-pixel redness of sclera (R) evaluation as follows:

(1) $$R=\frac{27}{4}\left(r-\sqrt{\frac{g^{2}+b^{2}}{2}}\right)\sqrt{r^{2}+g^{2}+b^{2}}{\left(1-\sqrt{r^{2}+g^{2}+b^{2}}\right)}^{2},$$

where *r, g,* and *b* are the normalized red, green, and blue intensities respectively. The mean redness and distribution of redness in the sclera are calculated for both the treated and nontreated eyes from all 3 eye positions. To allow for the inhomogeneity of the scleral reaction to the allergen (see Fig 1, *B*), the distribution of squared gradients of redness with 4 spatial steps was calculated.

Despite the use of a special mask (Fig 1, *A*), in some cases the changes of illumination in the room during the examination led to a shift of the white color balance of the images (compare the right and left images in Fig 1, *B*). To make our measurements robust to the illumination change, we used the sclera of the untreated (right) eye as an internal control. Toward this end, the redness distributions of both eyes were normalized (shifted) in such a way that the redness distribution of the right eye on the test and control images had a maximum possible overlap. After this normalization, the differences between the redness of the left eye in the test and control conditions were used as the AllergoEye scores.

### ROC curve

From these labels, we constructed a receiver operating characteristic (ROC) curve for AllergoEye, where for each threshold value we calculated

(2) $$Sensetivity=\frac{\text{true positive}}{\text{true positive}+\text{false negative}}$$

(3) $$Specificity=\frac{\text{true positive}}{\text{true positive}+\text{false positive}}.$$

### Statistical analysis

For significance estimation, we used the Student *t* test (∗*P* < .05; ∗∗*P* < .01; ∗∗∗*P* < .001). Statistical analysis and graph generation were performed by using model analysis software FitModel^23^ (http://pluk.mpi-cbg.de/projects/fitmodel).

## Results

**Fig 2:**
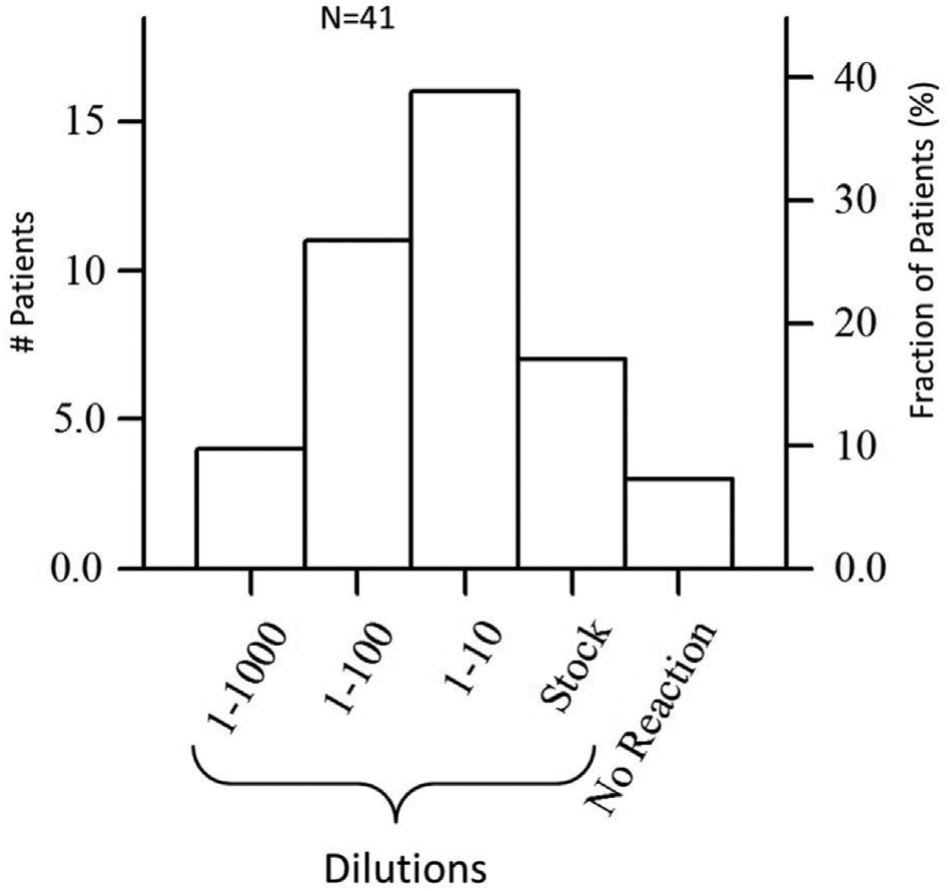
Distribution of the 41 study patients by sensitivity defined as allergen dilutions, with allergic reaction detected as an SSS of 3 or higher. Left axis is the number of patients; right axis is the percentage of patients.

First, we characterized the distribution of patients in the study by eye sensitivity to the grass allergen. As shown in Fig 2, most of the 41 patients who took part in the study (n =16 [∼40%]) exhibited a reaction at an allergen dilution of 1:10, whereas only 4 patients (10%) were sensitive to a dilution of 1:1000. Three patients (8%) did not display any eye reaction for provocation at highest concentration (stock solution) despite an anamnesis record of moderate-to-severe rhinoconjunctivitis. The number of CPT measurements per patient varied from 1 (obvious reaction on dilution 1:1000) to 4 (all dilutions up to stock solution), resulting in a total of 114 measurements from the 41 patients.

**Fig 3:**
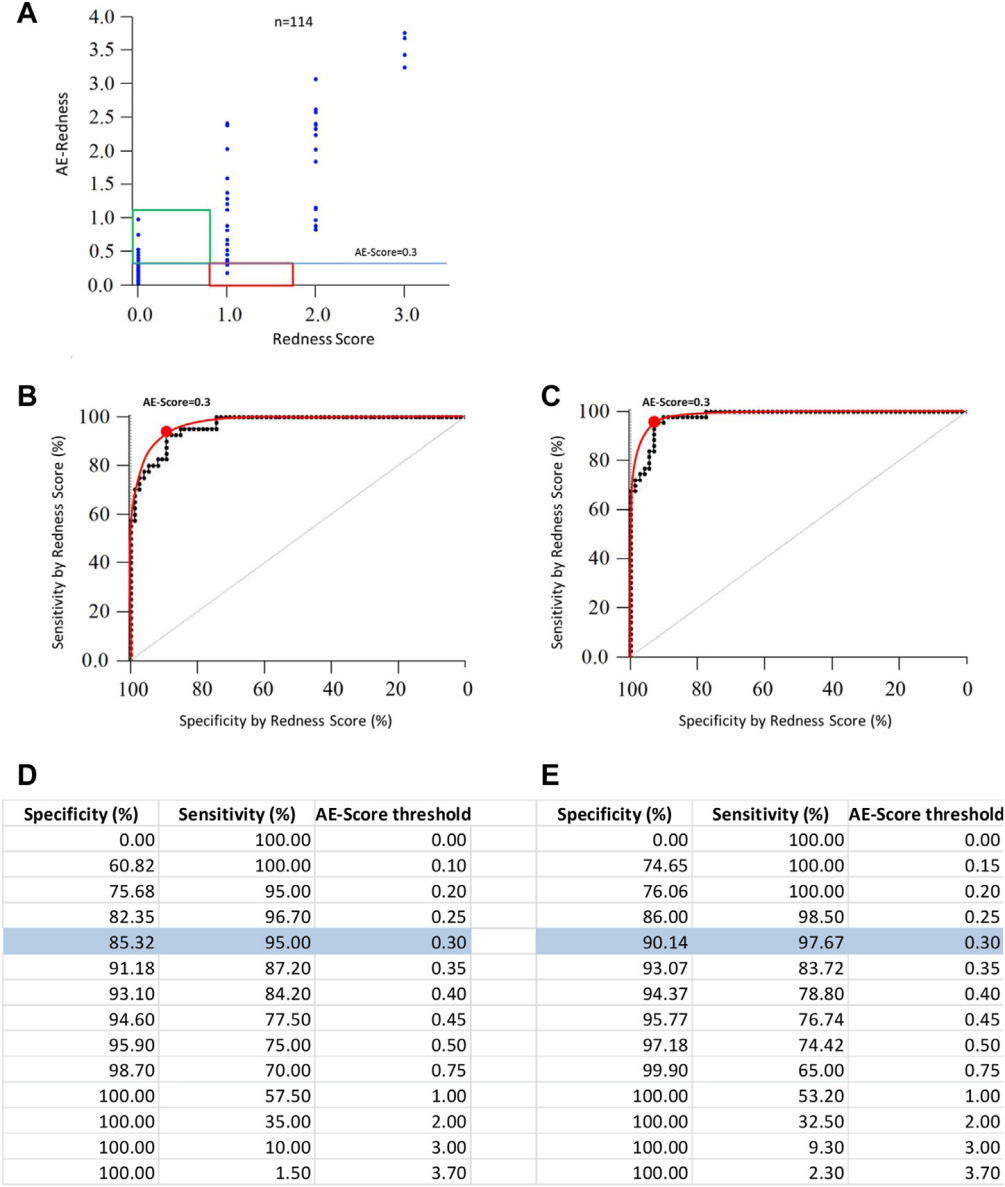
**A,** Scatterplot of 114 measurements (*blue dots*) that were made on 41 patients to reveal the dependence between redness score estimated by medical staff (*horizontal axis*) and redness evaluated by AllergoEye (*vertical axis*). Horizontal cyan line denotes a threshold value of 0.3 (see the text). Red and green rectangles highlight measurements that are false-negative and false-positive results, respectively. **B** and **D**, ROC curve (**B**) (sensitivity vs specificity), as defined by the threshold value gradually changing from 0.0 to 3.7 and its presentation in table form (**D**). Experimental data are represented as black dots; the interpolation curve is represented by a red line. The optimal threshold (0.3) is marked by a red dot. **C** and **E**, The ROC curve and its presentation in table form are similar to those in (**B**) and (**D**), but the measurements that are highlighted by red and green rectangles in (**A**) were reevaluated by 2 independent observers and the redness scores were corrected by majority voting (see Fig E1). Sensitivity and specificity at the optimal threshold value are highlighted in cyan in (**D**) and (**E**).

Next, we characterized AllergoEye’s sensitivity and specificity. Toward this end, we constructed a scatterplot for the 114 measurements (Fig 3, *A* [*blue dots*]). Each dot represents 1 measurement, where the x-value is a human-estimated redness score (range 0-3) and the y-value is an AllergoEye score (from 0 to 3.7). Then, we gradually changed the threshold value from 0 to 3.7, and for each threshold value we labeled the measurements as positive (above the threshold) or negative (below the threshold). As the ground truth labels we took the human-assigned scores. Therefore, a positive value was labeled as true positive if it corresponded to a human-assigned nonzero score; otherwise, it was labeled as false positive. The true- and false-negative labels were assigned in a similar way. From these labels, we constructed an ROC curve for AllergoEye, where for each threshold value we calculated equations (2) and (3).

The result is drawn as black dots on Fig 3, *B* and presented in the table that is Fig 3, *D*. The ROC curve was smoothed (red line in Fig 3, *B*); the optimal threshold value was found to be 0.3, as denoted by the red dot in the graph that is Fig 3, *B*, highlighted in the table that is Fig 3, *D,* and shown as a horizontal line in Fig 3, *A*.

We found that 15 AllergoEye measurements were labeled as false at the optimal threshold (red and green bars in Fig 3, *A* and Fig E1 in the Online Repository at www.jaci-global.org). The human-estimated scores of these measurements were reevaluated by 3 independent experts. Reevaluation changed the human-estimated scores in 9 cases (marked in green in Fig E1), whereas 6 were confirmed (marked in red in Fig E1). In summary, AllergoEye was found to be more accurate than the human operator (6 mistakes vs 9 mistakes, respectively). After reevaluation, a new ROC curve was constructed (Fig 3, *C* and *E*). From the table that is Fig 3, *E*, we found that AllergoEye's sensitivity to eye redness is as high as 97.7%, with 90% specificity.

**Fig 4:**
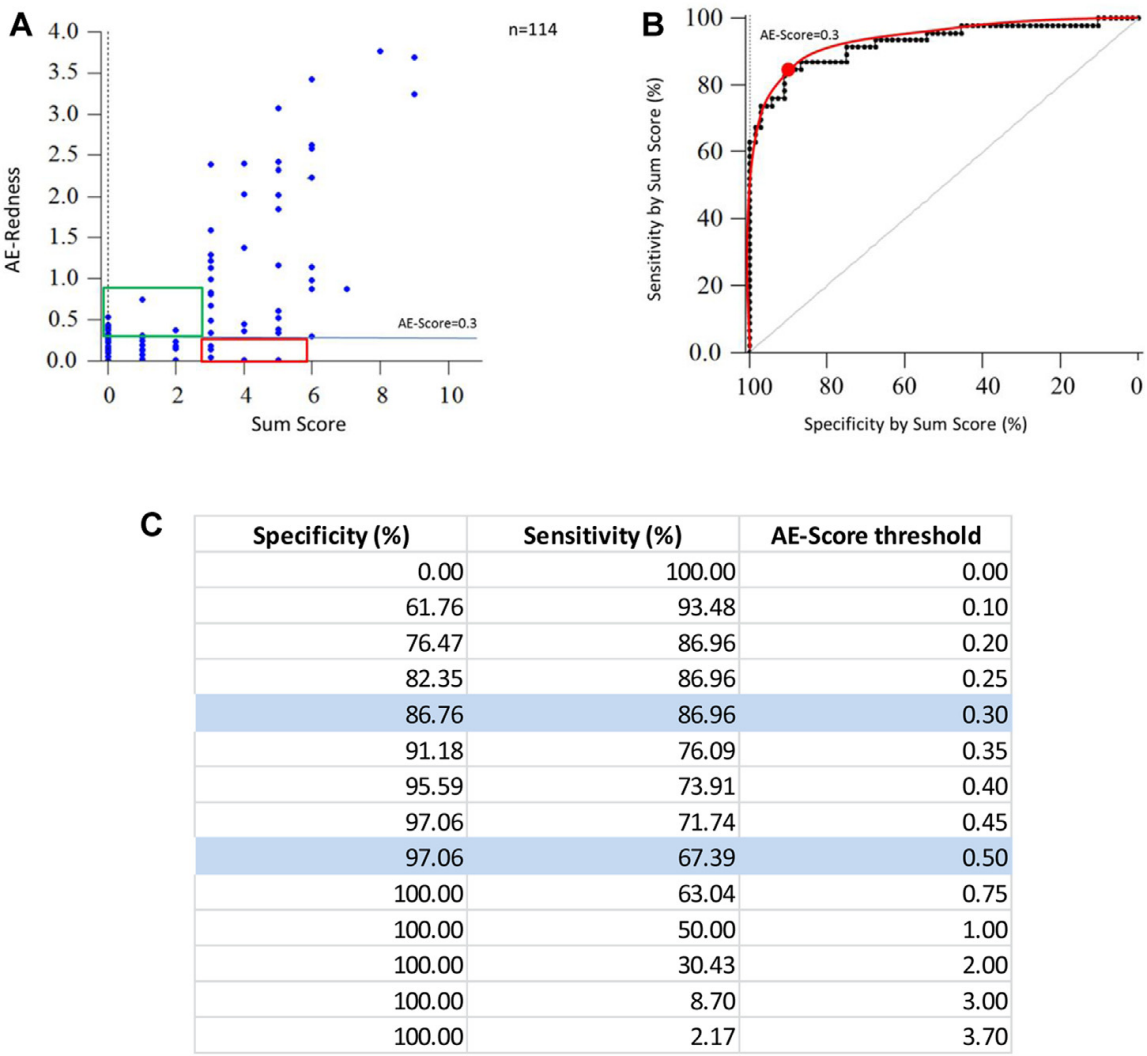
**A**, Scatterplot of 114 measurements (*blue dots*), which were made on 41 patients to reveal dependence between SSS (*horizontal axis*) and redness evaluated by AllergoEye (*vertical axis*). The horizontal cyan line denotes a threshold of 0.3. The red and green rectangles highlight measurements that are false-negative and false-positive results, respectively. **B** and **C,** The ROC curve (sensitivity vs specificity), as defined by the threshold value gradually changing from 0.0 to 3.7 (**B**), and its presentation in table form (**C**). The experimental data are represented as black dots; the interpolation curve is drawn by a red line. The optimal threshold (0.3) is marked by a red dot. Sensitivity and specificity at the optimal and stringent thresholds are highlighted in (**C**) in cyan.

Because an allergic reaction is usually estimated as the sum of subjective patient’s symptoms score and objective redness score^18^ (SSS), we decided to compare the AllergoEye score with SSS as a ground truth. The scatterplot of the 114 measurements with SSS scores (range 0-12) on the x-axis and the AllergoEye score (range 0-3.7) on the y-axis is drawn in Fig 4, *A* by using blue dots. Similar to Fig 3, *A* and *C*, the ROC curve was plotted in Fig 4, *B* and *C*. Interestingly, the optimal threshold value was found to be the same (0.3) as in the redness-only–based ground truth. However, the sensitivity and specificity were lower (∼87%) owing to the presence of non-redness–related subjective symptoms scores.

**Fig 5:**
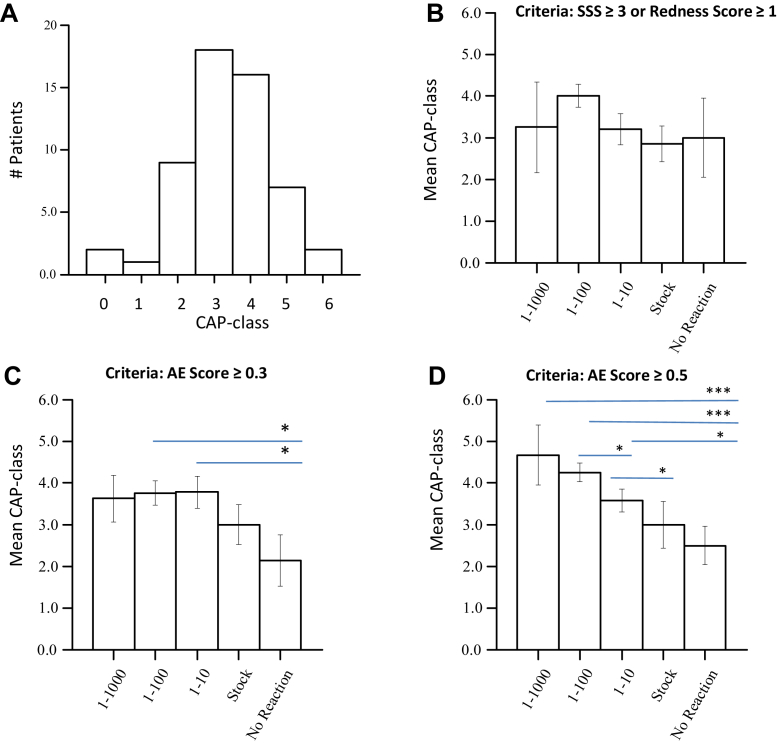
**A,** Distribution of the 41 patients by sIgE CAP classes. However, 2 patients have a score of CAP class 0 (their sIgE concentration was slightly below the lower boundary of CAP class 1) but have clinically relevant symptoms. **B,** Mean CAP class versus sensitivity to allergen dilutions, as determined by SSS. **C,** Mean CAP class versus sensitivity to allergen dilutions, as determined by AllergoEye with a cutoff threshold equal 0.3. **D,** Mean CAP class versus sensitivity to allergen dilutions, as determined by AllergoEye with a cutoff threshold equal 0.5. Asterisks denote statistically significant differences, with ∗*P* <.05; ∗∗*P* < .01; ∗∗∗*P* < .001.

To find the objectiveness of AllergoEye, we decided to compare patients' sensitivity as determined by the AllergoEye score with the sIgE concentration in their blood. The sIgE concentration was scored in terms of CAP classes.^24^ The characterization of the patients in the study by CAP classes is shown in Fig 5, *A*. Most of the patients have scores of CAP class 3 or CAP class 4; however, 2 patients have a score of CAP class 6, and 2 patients have a score of CAP class 0 (their sIgE concentration is slightly below the lower boundary of CAP class 1). We divided patients by sensitivity category according to dilutions at which the allergic reaction was detected according to criterion of an SSS of 3 or higher or a human-evaluated redness score higher than 1. For each category, the mean CAP classes were calculated. In line with previous reports,^25^^,^^26^ we did not find a significant correlation between mean CAP classes and patients' allergic sensitivity, as measured by SSS (Fig 5, *B*). Next, we constructed graphs for mean CAP classes versus AllergoEye-evaluated allergy sensitivity for the threshold value of 0.3 (Fig 5, *C*) and threshold value of 0.5 (Fig 5, *D*). Surprisingly, even with an AllergoEye threshold value of 0.3, we found statistically a significant difference between the CAP classes of sensitive and insensitive patients (Fig 5, *C* [*asterisks*]). With an AllergoEye threshold value of 0.5 (which corresponds to a specificity of 97% for SSS [Fig 4, *C*]), we uncovered a statistically significant relationship between CAP classes and patients' allergy sensitivity (Fig 5, *D* [*asterisks*]).

## Discussion

The quantitative analysis of CPT in clinical practices is a long-standing challenge in allergology. Thus far, most attempts to quantify the results of the CPT have been based on the quantitative analysis of eye reactions to an allergen in the manually segmented region of interest (ROI) in the sclera, either by measuring the apparent area of vessels^4^^,^^12^ or by per-pixel evaluation of redness.^4^^,^^14^ Although successful in clinical studies, the methods based on manual ROI selection were too labor-intensive for wide-scale practice, and methods based on the apparent area of vessels were insensitive to the diffuse redness reaction of the sclera. To the best of our knowledge, the article by Sirazitdinova et al is the only work published to date in which automated sclera segmentation has been implemented (by the random forest method).^14^ However, similar to the procedure used in previous works,^4^^,^^12^^,^^15^^,^^18^ the method of Sirazitdinova et al^14^ requires matching automatically selected key points on the sclera before and after provocation, which makes it too complicated for use in general practice. Indeed, such matching requires an accurate repetitive eyeball orientation from the patient during image acquisition, which significantly complicates the process of measurement, and in the event of operator carelessness, it could lead to a significant degradation of CPT quantification accuracy.

Further, measurement of the sclera's redness is also impeded by the variation in illumination and white balance in digital cameras. During the allergen titration time, which can take tens of minutes, the environment illumination conditions could change. Additional devices such as a face mask are very useful, but unfortunately, they do not allow total suppression of the fluctuations in white balance. None of the previously proposed methods handle the changes in environment illumination conditions in an automated manner.^4^^,^^12^^,^^14^^,^^15^

We have developed a method that uses (1) a deep neural network (33 layers) for automated segmentation of iris and sclera on the image, which excludes manual ROI selection and as such increases the throughput of the method; (2) a new formula (equation 1) for brightness-independent evaluation of redness per pixel; (3) a sclera redness score based on pixels' redness distribution that does not require key point matching before and after provocation; and (4) an automated white balance correction based on the untreated eye as an internal (in-image) control that makes the method robust to changes in environment illumination conditions. It is worth repeating that our method of evaluating allergic reaction is not based solely on widening macroscopically recognizable vessels but also captures changes in the capillary network that manifest itself in the diffuse redness of the sclera.

Nowadays, the criterion standard of rating the results of CPTs is the so-called SSS, although there are more than 1 score definitions in use today.^18^ However, the SSS includes patients' subjective feelings and fails to reveal a correlation with titers of sIgE, as was previously reported in literature,^25^^,^^26^ in which a correlation was found for very high sIgE titers and high SSS only. Such an absence of correlation between CAP classes and SSS was confirmed in our study (Fig 5, *B*). One possible explanation for the absence of a correlation between SSS and CAP classes for moderately sensitive patients may be the personal ability to tolerate unpleasant feelings, which differs between patients and thus results in wide variation in subjective score values. Therefore, SSS-based methods only partially satisfy the requirement for evidence-based medicine, in which especially accurate numeric values are essential in clinical studies. In contrast, the AllergoEye scores revealed an evident correlation between sIgE CAP classes and patients’ sensitivity to allergen provocation (Fig 5, *D*). Therefore, AllergoEye scores alone, as well as those incorporated into the SSS,^18^ could provide an objective method for clinical studies and control therapy in general allergology practice.

Validating diagnostic methods in medicine is an essential task in new developments. The main objective of this study was validation of the AllergoEye system by determining the sensitivity and specificity curve, the so-called ROC curve, and the selection of optimal and strict thresholds for evaluation of allergic response. This validation was based on 2 considerations. First, we compared the dilution of the CPT solution that triggered a reaction as detected by the redness determined by medical staff with the redness detected by AllergoEye (Fig 3, *B* and *C*). Second, we compared the dilution of the CPT solution that triggered a reaction as detected by the redness determined by the SSS with the redness detected by AllergoEye (Fig 4, *B*). Both comparisons showed the high sensitivity and specificity of AllergoEye (Figs 4, *B-D* and 5, *B* and *C*) as an instrument for redness and allergy assessment. In addition, the superiority of AllergoEye over human- and SSS-based estimation in terms of accuracy of obtained results (in terms of false-positive and false-negative results) was clearly demonstrated.

Selecting the right patients for clinical trials in allergology overall and also using CPT is an outstanding problem. The challenge lies in the large variability of the subjective symptoms of CPT and absence of a clear correlation between these symptoms and objective parameters, in particular the sIgE titer. On the basis of the AllergoEye ROC curves, the required specificity can be chosen from the tables presented in Figs 4 and 5. The strict specificity allows objective selection of high-sensitivity patients. At the same time, the sensitivity value allows estimation of the number of patients that must be screened, which is essential for clinical study design. In a clinical trial, AllergoEye could be used for quantitative monitoring of the efficiency of allergy therapy (eg, hyposensitization).

This clinical validation of the AllergoEye method on patients with grass allergy was successful and demonstrates the usefulness for a wider range of routine examinations and clinical studies in allergology.

### Conclusion

At the optimal threshold AE-score of 0.3, AllergoEye, an AI-based platform for the quantification of CPT results, showed high sensitivity and specificity compared with both the the score of redness of eye, as measured by medical personnel (RMS) (> 95%) and the SSS score, which represents subjective patient reports (>86%). It was also shown that a higher threshold AE-score of 0.5 the method has a specificity greater than 0.97%, which results in clear and statistically significant correlation of the invoked allergic reaction in the eye with the IgE CAP classes in the blood. We found that the optimal threshold of 0.3 could be recommended for immune therapy control, whereas the high-specificity threshold of 0.5 is better suited for selecting patients for clinical studies. Clinical validation demonstrated that AllergoEye is a sensitive and efficient instrument for objective evaluation of allergic reactions. It could be used for patient selection and control of the treatment efficiency in clinical studies as well as for diagnostic and therapy control in routine allergology practice.

## Disclosure statement

Disclosure of potential conflict of interest: The authors declare that they have no relevant conflicts of interest.
