# Supplementary material for: Validation of AI-based software for objectification of conjunctival provocation test
Source: J Allergy Clin Immunol Glob. 2023 May 30;2(3):100121. doi: 10.1016/j.jacig.2023.100121 (PMC10509841; doi:10.1016/j.jacig.2023.100121)
Supplement: Supplementary data [file mmc1.pdf]

Suppl. Fig.1

Gras 1-100	Redness was re-evaluated and found positive	TP
Gras 1-1 000	Redness was re-evaluated and found negative	FP
Gras 1-100	Redness was re-evaluated and found negative	FP
Gras 1-1 000	Redness was re-evaluated and found positive	TP
Gras 1-1 000	Redness was re-evaluated and found positive	TP
Gras 1-100	Redness was re-evaluated and found negative	TN
Gras 1-1 000	Redness was re-evaluated and found negative (red eye on start) (**CAP-class =1**)	FP
Gras 1-10	Redness was re-evaluated and found negative	TN
Gras 1-100	Redness was re-evaluated and found negative	TN
Gras 1-100	Redness was re-evaluated and found positive	TP
Gras 1-10	Redness was re-evaluated and found positive	FN
Gras Stocklösung	Redness was re-evaluated and found negative	TN
Gras 1-1 000	Redness was re-evaluated and found positive	TP
Gras 1-1 000	Redness was re-evaluated and found negative	FP
Gras 1-1 000	Redness was re-evaluated and found positive (**CAP-class = 0**)	FN

Suppl. Fig.2

	CAP Class						
	0	1	2	3	4	5	6
Male	1	1	3	8	10	1	1
Female	1	0	5	5	2	6	1

Suppl. Fig.3

Itching	
☐	0 = Not itchy
☐	1 = Slightly itchy but not bothersome
☐	2 = Itchy, bothersome and/or urge to rub eye at times
☐	3 = Very itchy, very bothersome and/or urge to rub eye often
Irritation	
☐	0 = Not irritated
☐	1 = Dry eye or other unusual feeling in eye, but not bothersome
☐	2 = Gritty or foreign object feeling in eye, bothersome and/or urge to rub eye
☐	3 = Burning or painful eye, very bothersome and/or difficulty keeping eye open and/or urge to rub eye often
Tearing	
☐	0 = Not watery
☐	1 = Watery eye, but no tears and not bothersome
☐	2 = Watery eye with a few tears, bothersome and/or urge to wipe eyes at times
☐	3 = Watery eye with several tears, very bothersome and/or urge to wipe eye often
Redness	
☐	0 = No redness
☐	1 = Redness in conjunctiva bulbi
☐	2 = Redness in conjunctiva bulbi and tarsi
☐	3 = Redness in conjunctiva bulbi and tarsi and edema
