# Supplementary material for: Validation of AI-based software for objectification of conjunctival provocation test
Source: J Allergy Clin Immunol Glob. 2023 May 30;2(3):100121. doi: 10.1016/j.jacig.2023.100121 (PMC10509841; doi:10.1016/j.jacig.2023.100121)
Supplement: Supplementary Figure [file mmc2.docx]

**Supplemental Figure Legend**

**Suppl. Figure 1**. Table of re-evaluation of eye redness as defined by medial staff during CPT for the measurements highlighted on Figure 3A. The redness scores that were kept unchanged is highlighted by red, those that were changed after re-evaluation are highlighted by green. The re-evaluation was performed by 2 independent observers and final decision was done by majority vote.

**Suppl. Figure 2**. CAP-classes distribution among patients.

**Suppl. Figure 3**. Example of symptom score evaluation protocol.
